# The outcomes of right and left complicated colonic diverticulitis

**DOI:** 10.1016/j.sopen.2025.06.005

**Published:** 2025-06-15

**Authors:** Anh Tuan Nguyen, Quang Tien Pham, Hoi Van Tran, Hoang Viet Truong, Loc Huynh Tran

**Affiliations:** a Gia Dinh People's Hospital, Binh Thanh District, Ho Chi Minh City, Viet Nam; b University of Medicine and Pharmacy at Ho Chi Minh City, Viet Nam

**Keywords:** Colon diverticulitis, Complication, Risk factor

## Abstract

**Background:**

The incidence of complicated colonic diverticulitis is increasing in Asia, with notable differences in management between right-sided (RCD) and left-sided (LCD) cases. This study compared treatment outcomes and identified risk factors for complications.

**Methods:**

A retrospective analysis was performed on 181 patients diagnosed with complicated colonic diverculitis from January 2022 to September 2024, including 99 RCD and 82 LCD cases.

**Results:**

The mean age in the RCD group was 43.31 ± 14.6 years, compared to 63.9 ± 12.9 years in the LCD group. Recurrence rates were higher in LCD than RCD (19.5 % vs. 7.1 %). Surgical intervention was more frequent in LCD cases (63.4 % vs. 9.2 %), with longer hospital stay (9.3 vs 4.9 days). All RCD perforations were managed with primary anastomosis. Hartmann's procedure was the most common approach for LCD, with primary resection and anastomosis performed in 26.9 %. Postoperative complications occurred in 27.8 % of LCD patients, including wound infections, intra-abdominal abscesses, and pneumonia. Three patients in the LCD group died during hospitalization. Fever, elevated CRP levels, surgery type, and prolonged hospital stays were independent risk factors.

**Conclusion:**

Patients with complicated RCD were younger than those with LCD. Conservative treatment for RCD had a high success rate, while complicated LCD often required surgery due to peritonitis. Fever, CRP level, type of surgery, and hospital stay were independent risk factors for complications.

## Graphical abstract

**Figure:**
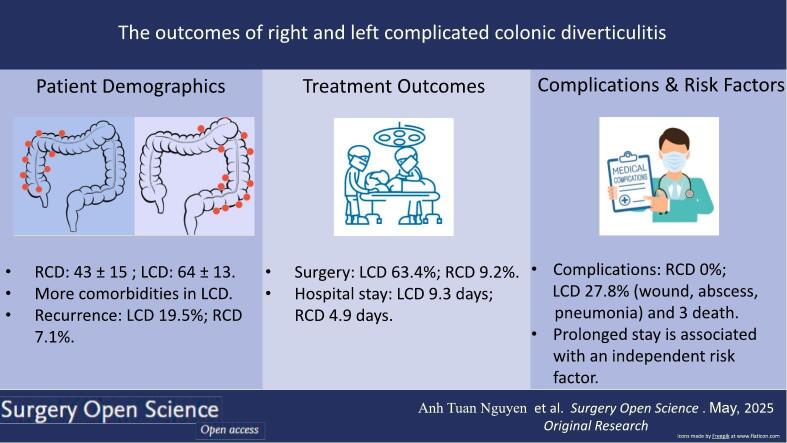

## Introduction

Colonic diverticulitis is a common surgical condition primarily seen in Western countries, where most cases involve left-sided colonic diverticulitis (LCD) [1]. In Asia, right-sided colonic diverticulitis (RCD) is more common [2,3]. However, the number of LCD cases has increased in recent years. In Japan, while 70.1 % of colonic diverticulitis cases are RCD, 57.5 % of complicated cases occur on the LCD [4]. According to Chung (2016), conservative medical treatment for RCD has a high success rate (94 %). In contrast, among the 35 LCD cases studied, 65.7 % were complicated and required emergency surgery [5].

The treatment of LCD followed the guidelines of the World Society of Emergency Surgery (WSES). However, there are no specific guidelines for RCD, which is often treated using the same classification and approach as LCD [6].

The decision between medical treatment and surgical intervention depends on the location of the diverticulitis and severity of the complications. Factors such as patient age, BMI, and pre-existing health conditions also play a significant role in determining treatment outcomes [5]. This study aimed to evaluate the treatment outcomes for two groups of patients with complicated LCD and RCD and to identify the risk factors associated with complications.

## Methods

Data of all patients diagnosed with complicated diverticulitis between January 2022 and September 2024 at the Department of Gastrointestinal Surgery, Gia Dinh People's Hospital, were retrospectively collected and analyzed. Patients were excluded if they did not undergo MSCT before diagnosis, had diverticulitis affecting both the left and right sides, underwent surgery without postoperative pathology confirming diverticulitis, or had a prior history of gastrointestinal cancer. Complicated diverticulitis was classified as grade Ia or higher based on the WSES classification, which is applicable to both RCD and LCD cases [6]. RCD was defined as diverticula located from the cecum to the right transverse colon, whereas LCD was defined as diverticula from the remaining transverse colon to the sigmoid colon.

Data extracted from medical records included age, sex, comorbidities, body mass index (BMI), history of diverticulitis, clinical symptoms, location and severity of complications (using the WSES and Hinchey classifications), surgical methods, and postoperative complications categorized by the Clavien-Dindo system. The risk factors associated with complications were recorded in both the right- and left-sided groups. Patients who required readmission within 30 days of discharge for medical or surgical reasons were classified as treatment failures. This study was approved by the Ethics Committee of the Gia Dinh People's Hospital.

Data were analyzed using the SPSS Statistics 27.0. Categorical variables were analyzed using Chi-square and Fisher's exact tests, and continuous variables were assessed using Student's *t*-test. Logistic regression was used for multivariate analysis to identify risk factors associated with morbidity and mortality. Statistical significance was set at P < 0.05.

## Results

A total of 405 patients were diagnosed with colonic diverticulitis, including 282 patients with RCD and 123 patients with LCD. After excluding patients without perforation complications, 181 patients with complicated colonic diverticulitis were analyzed. Complication rates for RCD and LCD were 99 patients (35.1 %) and 82 patients (66.6 %), respectively. The clinical characteristics are summarized in Table 1.

**Table 1:** Patient demographics of perforated colonic diverticulitis in both sides.

	Total N = 181	RCD N = 99	LCD N = 82	p. value
Age, yr	52.59 ± 17.5	43.31 ± 14.64	63.79 ± 13.76	p < 0.001
<40	46 (25.4 %)	42 (42.5 %)	4 (4.9 %)	
40–70	107 (59.1 %)	54 (54.5 %)	53 (64.6 %)	
>70	28 (15.5 %)	3 (3 %)	25 (30.5 %)	
Gender (M/F)	107/74	68/31	39/43	p = 0.004
	(59.1 %/ 40.9 %)	(68.7 %/ 31.3 %)	(47.6 %/ 52.4 %)	
Comorbidity				
Hypertension	57 (31.5 %)	13 (13.1 %)	44 (53.7 %)	p < 0.001
Diabetes	28 (15.5 %)	3 (3.0 %)	25 (30.5 %)	p < 0.001
COPD	3 (1.7 %)	1 (1 %)	2 (2.4 %)	p = 0.59⁎
Others	28 (15.5 %)	10 (10.1 %)	18 (22 %)	p = 0.02
BMI	22.98 ± 3.50	23.04 ± 3.38	22.91 ± 3.65	p = 0.80
<18,5	11 (6.1 %)	3 (3 %)	8 (9.8 %)	
18,5–25	121 (66.9 %)	70 (70.7 %)	51 (62.2 %)	p = 0.58
>25	49 (27.1 %)	26 (26.3 %)	23 (28 %)	
Previous history of acute diverculitis	23 (12.7 %)	7 (7.1 %)	16 (19.5 %)	p = 0.01
Fever >38 °C	19 (10.5 %)	6 (6.1 %)	13 (15.9 %)	p = 0.03
Rebound tendeness	61 (33.7 %)	27 (27.3 %)	34 (41.5 %)	p = 0.04
WBC count	13.79 ± 4.94	13.30 ± 4.41	14.39 ± 5.47	p = 0.145
CRP	93.23 ± 91.22	86,80 ± 73.24	99.08 ± 108.93	p = 0.38
WSES classification				p < 0.001
Ia	98 (54.1 %)	75 (75.8 %)	23 (28 %)	
Ib	28 (15.5 %)	14 (14.1 %)	14 (17.1 %)	
IIa	25 (13.8 %)	8 (8.1 %)	17 (20.7 %)	
IIb	14 (7.7 %)	2 (2 %)	12 (14.6 %)	
III	8 (4.4 %)	0	8 (9.8 %)	
IV	8 (4.4 %)	0	8 (9.8 %)	
Abscess drainage	5 (2.8 %)	3 (3 %)	2 (2.4 %)	P = 1.0
Surgery	61 (33.9 %)	9 (9.2 %)	52 (63.4 %)	p < 0.001
Hospital stay (days)	6.92 ± 5.40	4.94 ± 2.38	9.30 ± 6.89	p < 0.001

⁎ Fisher's Exact test are used.

The mean age in the RCD group was 43.31 ± 14.6 years, while the LCD group was 63.9 ± 12.9 years. Patients in the RCD group were significantly younger than those in the LCD group (p < 0.001). Patients in the LCD group were predominantly aged 40 years and older, whereas RCD occurred across a broader age range, with a tendency to affect younger individuals. The proportion of males with complications was higher in the RCD group than in the female group, whereas in the LCD group, the ratio was equal, with a statistically significant difference. Chronic conditions such as hypertension and diabetes mellitus were more common in the LCD group (p < 0.001). Recurrence rates were significantly higher in the LCD group than in the RCD group (p = 0.01). None of the RCD group had complications of grade III or higher according to the WSES classification, while 34.2 % of the LCD group had complications of grade IIb or higher compared to only 2 % in the RCD group. Surgical intervention was required much more frequently in the LCD group (63.4 %) than in the RCD group (9.2 %), and the hospital stay for LCD was longer (9.3 vs 4.9 days), with statistically significant differences compared to the RCD group.

In terms of treatment, percutaneous abscess drainage was performed in only five patients (2.4 %), and none of these required surgery afterward. Surgical treatment outcomes were recorded in 61 patients (33.9 %) and are detailed in Table 2.

**Table 2:** Surgical treatment in the 2 groups.

	Total	RCD	LCD	p. value
Operation performed	61 (100 %)	9 (14.7 %)	52 (85.3 %)	p < 0.001
Laparocopy	22 (36.1 %)	5 (55.6 %)	17 (32.7 %)	p = 0.263
Laparotomy	39 (63.9 %)	4 (44.4 %)	35 (67.3 %)	
Hinchey classification				p = 0.018⁎
Hinchey I	10 (16.4 %)	3 (33.3 %)	7 (13.5 %)	
Hinchey II	23 (37.7 %)	6 (66.7 %)	17 (32.7 %)	
Hinchey III	22 (36.1 %)	0 (0 %)	22 (42.3 %)	
Hinchey IV	6 (9.8 %)	0 (0 %)	6 (11.5 %)	
Surgical procedure:				p < 0.001⁎
Hartmann's procedure	33 (54.1 %)	0 (0 %)	33 (63.5 %)	
Primary resection anastomosis	23 (37.7 %)	9 (100 %)	14 (26.9 %)	
Simple suture and lavage	3 (4.9 %)	0 (0 %)	3 (5.8 %)	

⁎ Fisher's Exact test are used.

All surgical cases of RCD involved primary resection and anastomosis. In contrast, Hartmann's procedure was the most frequently performed surgery for LCD, with primary resection and anastomosis conducted in only 14 LCD patients (26.9 %). Additionally, simple suturing with peritoneal lavage was performed in 3 LCD patients due to intraoperative findings of small perforations (Hinchey III classification) and stable hemodynamic conditions.

Postoperative complications occurred in 17 LCD patients (27.8 %) who underwent surgery. Among these, 14 patients experienced mild complications (Clavien-Dindo grades I and II), while three patients developed severe complications, including respiratory failure leading to death in two patients and severe septic shock in one patient (Clavien-Dindo grades IVb and V). Mild complications were managed with medical treatment, and none of the patients required postoperative intra-abdominal abscesses (Table 3). In contrast, no postoperative complications were reported in the RCD group during hospitalization.

**Table 3:** Postoperative complications of perforated colonic diverticulitis.

	RCD N = 9	LCD N = 52
Wound infection	0	5 (8.2 %)
Intra-abdominal abscesses	0	1 (1.6 %)
Pneumonia	0	11 (18.0 %)
Death	0	3 (4.9 %)

Factors such as age, gender, chronic diseases, BMI, WBC count, preoperative WSES classification, intraoperative Hinchey classification, surgical procedure and time of surgery did not significantly influence treatment complications. Multivariate analysis identified fever at admission, elevated CRP levels, the type of operation performed, and prolonged hospital stays as independent risk factors significantly associated with treatment complications (Table 4).

**Table 4:** Risk factors for morbidity and mortality after surgery in patients with left-sided colonic diverticulitis.

	OR	95 % Cl	p. value
Age	1.01	0.96–1.06	0.76
Gender	1.16	0.33–4.08	0.81
Comorbidity	1.06	0.29–3.85	0.92
BMI	0.92	0.17–1.10	0.39
Fever	1.9	0.75–4.78	0.17
WBC	0.89	0.79–1.02	0.09
CRP	1.0	0.99–1.00	0.64
Time of surgery	1.00	0.99–1.01	0.1
Hospital stay	1.27	1.09–1.47	0.001

## Discussion

Colonic diverticulosis is a common condition worldwide. In Europe, LCD accounts for 90 % of cases [7], while in Asia, RCD is more preveland, with rates of 85 % in South Korea [8] and 70.1 % in Japan [4]. Approximately 25 % of patients with colonic diverticulosis develop diverticulitis, with diverticular perforation being the most common complication, occurring in 12 % of the cases. Perforation allows pus or fecal matter to leak into the abdominal cavity, causing peritonitis [9]. In our study, RCD was 2.3 times more common than LCD, consistent with findings from other studies in Asia [10,11]. However, complications from left-sided perforations are nearly twice as common as those from right-sided perforations. RCD was more frequently observed in younger male patients, whereas LCD was more common in older female patients. Manabe reported that 44.2 % of the patients with comorbidities such as cerebrovascular disease, respiratory disease, diabetes, and renal failure were at a significantly higher risk of complications than those without these complications [4]. Comorbidities, which are more common in older patients, explain why LCD patients in our study had higher rates of hypertension and diabetes than RCD patients (p < 0.001). Other risk factors for complicated diverticulitis include physical inactivity, obesity, constipation, and smoking [12]. However, no significant difference in BMI was found between the two groups in our study.

We found that the recurrence rate of LCD was higher than that of RCD (P = 0.01). Tsang and Chung also reported similar results, with recurrence rates of 9.9 % vs. 8.3 % [11] and 11.4 % vs. 9.6 % [5], respectively, with no statistically significant difference between the two groups. However, a recent systematic review and meta-analysis involving 10.129 patients showed an overall recurrence rate of 10 % (95 % CI, 8–13 %; p < 0.01) for RCD, which was significantly lower than the 20 % recurrence rate (95 % CI, 16–24 %; p < 0.01) for LCD [13]. Fever at admission is a clinical symptom that often indicates complications. Manabe compared patients with and without diverticulitis complications and found that fever was significantly more common in the complicated group than in the non-complicated group (51.4 % vs. 14.1 %; p < 0.001) [4]. Tsang et al. reported that fever was more frequent in patients with perforated LCD than in those with perforated RCD (47.2 % vs. 31.7 %; p < 0.05) [11]. However, Chung and Lee did not find differences in fever between right- and left-sided diverticulitis, regardless of the present of complications [5,10]. In our study, only 10.5 % of patients had fever, which was significantly more common in perforated LCD cases (15.9 %) than in RCD cases (6.1 %). Fever was identified as an independent risk factor for treatment complications.

Patients with perforated RCD often present with localized pericolonic air or abscesses, rather than more severe diffuse or fecal peritonitis. According to the WSES guidelines, conservative treatment involving antibiotics and abscess drainage is recommended [6]. In our study, we prioritized this approach by combining antibiotic therapy with abscess aspiration and achieved a high success rate of 90.8 %. A retrospective study involving 167 patients with RCD reported that 12 patients (7 %) had complications. Among these, 2 required emergency surgery due to peritonitis and sepsis, 3 had uncomplicated cases, and 5 had perforations that did not respond to conservative management and underwent right colectomy [5]. In our study, right hemicolectomy was performed in 9 patients due to recurrent diverticulitis or abscesses located in areas difficult to drain. These patients were classified as Hinchey stage I or II during surgery, allowing for primary resection and anastomosis. No postoperative complications were observed in any of the patients. In contrast, perforated LCD typically present with more severe clinical manifestations. Many cases were accompanied by peritonitis, resulting in a significantly higher rate of surgical intervention (63.4 %) than in perforated RCD (p < 0.001). During surgery, 53.8 % of LCD cases involved peritonitis caused by pus or feces (Hinchey grades III and IV), reflecting disease severity. This condition increases the risk of mortality, leading to higher complication rates and a prolonged hospital stay. Tsang et al. reported that patients with perforated LCD had a significantly higher overall rate of treatment complications than those with RCD (51.5 % vs. 10.4 %; p < 0.001). Among the LCD patients, 21.2 % experienced medical complications, including pneumonia (4 patients), myocardial infarction (1 patient), and pulmonary embolism (4 patients). Surgical complications occurred in 36.4 % of the LCD cases, including surgical site infections (9 patients), anastomotic leaks (1 patient), and residual abscesses (4 patients). The average hospital stay for patients with LCD was longer than that for patients with RCD (5 days vs. 4 days; p < 0.001) [11].

Surgical methods for treating diverticulitis were chosen based on the patient's condition, including Hartmann's procedure, primary anastomosis, and simple suturing with abdominal lavage. Hartmann's procedure is commonly used because it avoids the risks associated with creating anastomosis in patients with peritonitis. Tsang's study involving 142 patients with perforated LCD, Hartmann's procedure was the most frequently performed surgery (54.5 %), followed by primary anastomosis (21.2 %), and abdominal lavage (18.1 %) [11]. Similar results were observed in a cohort study of 3.873 patients, in which Hartmann's procedure accounted for 64 % of the cases, including both open and laparoscopic surgeries [14]. In our study, Hartmann's procedure was the primary choice of surgery. Primary anastomosis was reserved for patients with hemodynamic stability, mild infections, and well-controlled comorbidities. The choice of surgical method depends on various factors, including the severity of peritonitis, patient's hemodynamic stability, presence of infection or sepsis, organ dysfunction, comorbidities, and the surgeon's expertise [15]. Nascimberi suggested using the qSOFA score at admission and the Mannheim Peritonitis Index (MPI) during surgery to guide the choice between Hartmann's procedure and primary anastomosis. Patients with qSOFA score < 2 and MPI < 21 were considered candidates for primary anastomosis [16,17]. Meta-analyses of RCTs showed no significant differences in complications or mortality between the two methods in stable Hinchey III and IV cases. However, observational studies found that primary anastomosis was associated with lower rates of complications (OR = 0.67; p = 0.02) and mortality (OR = 0.46; p < 0.001) than Hartmann's procedure [18]. The LADIES trial, which compared 130 patients with perforated LCD (Hinchey III and IV), found that 1-year survival was higher in the primary anastomosis group than in Hartmann's procedure group (HR = 2.79; p < 0.001) [19]. In our study, patients selected for primary anastomosis typically had a qSOFA score ≤ 1, Hinchey grade I–III, MPI < 21, and stable comorbidities. However, the surgeon's experience and patient's specific condition also played a significant role in determining the surgical approach.

The overall mortality rate of complicated diverticulitis was approximately 10.64 % [20]. In Asia, mortality from perforated RCD is very low (<1 %) [4,10,11], whereas perforated LCD has higher mortality rates, reported at 4.9 % and 4.2 % in different studies [4,11]. In Europe, a retrospective study by Hussain on 110 patients with complicated perforated diverticulitis reported an overall mortality rate of 14.53 %, with 12 deaths (10.9 %) attributed to perforation-related complications, including one intraoperative death, eight postoperative deaths, and three deaths without surgery [21]. An analysis of 993.220 patients found a mortality rate of 5.4 % for complicated perforated diverticulitis. Patients who underwent surgery had significantly higher mortality rates than those who did not (6.3 % vs. 3.0 %; p < 0.001) [22]. The difference in mortality rates for perforated LCD between Asian and Western countries was minimal.

The risk factors for mortality vary between regions. In Western countries, these include younger age, male sex, obesity, smoking, recurrent diverticulitis, comorbid chronic diseases, and delayed surgical [21,22]. In Asia, mortality risks are higher in older age, fever, left-sided diverticulitis, and delayed hospital admission [4,10]. These differences may reflect genetic and environmental factors. A meta-analysis of 59 global studies (1980–2012) identified factors contributing to postoperative mortality, including emergency versus elective surgery (OR = 6.12; p = 0.008), open versus laparoscopic surgery (OR = 36.43; p < 0.001), and Hartmann's procedure versus primary anastomosis (OR = 25.45; p < 0.001) [20]. Our study also found that fever, elevated CRP levels, open surgery, and prolonged hospital stay were significant risk factors for complications and mortality. These findings emphasize the need for effective infection control, timely surgical intervention, careful selection of surgical techniques, and improved postoperative care to reduce adverse outcomes.

Our study is a retrospective review of medical records, leading to certain limitations, such as incomplete data on diet, smoking, and timing of surgical intervention. Additionally, treatment and surgical decisions depended heavily on the surgeon's experience, with no standardization in practice, and no long-term follow-up of the patients. These limitations should be addressed in future studies to generate more comprehensive data for improving patient management.

## Conclusion

Conservative treatment for complicated RCD is highly successful. Complicated LCD often present with severe infections that require surgery, particularly peritonitis. Key factors, such as fever, elevated preoperative CRP levels, open surgery, and prolonged hospital stay, significantly increase the risk of complications and mortality during treatment.

## CRediT authorship contribution statement

**Anh Tuan Nguyen:** Writing – review & editing, Writing – original draft, Conceptualization. **Quang Tien Pham:** Writing – review & editing. **Hoi Van Tran:** Writing – review & editing, Investigation. **Hoang Viet Truong:** Investigation. **Loc Huynh Tran:** Investigation.

## Ethical approval statement

The research reported has adhered to relevant ethical guidelines.

## Funding

There is no funding to report for this study.

## Declaration of competing interest

The authors report no conflicts of interest.
